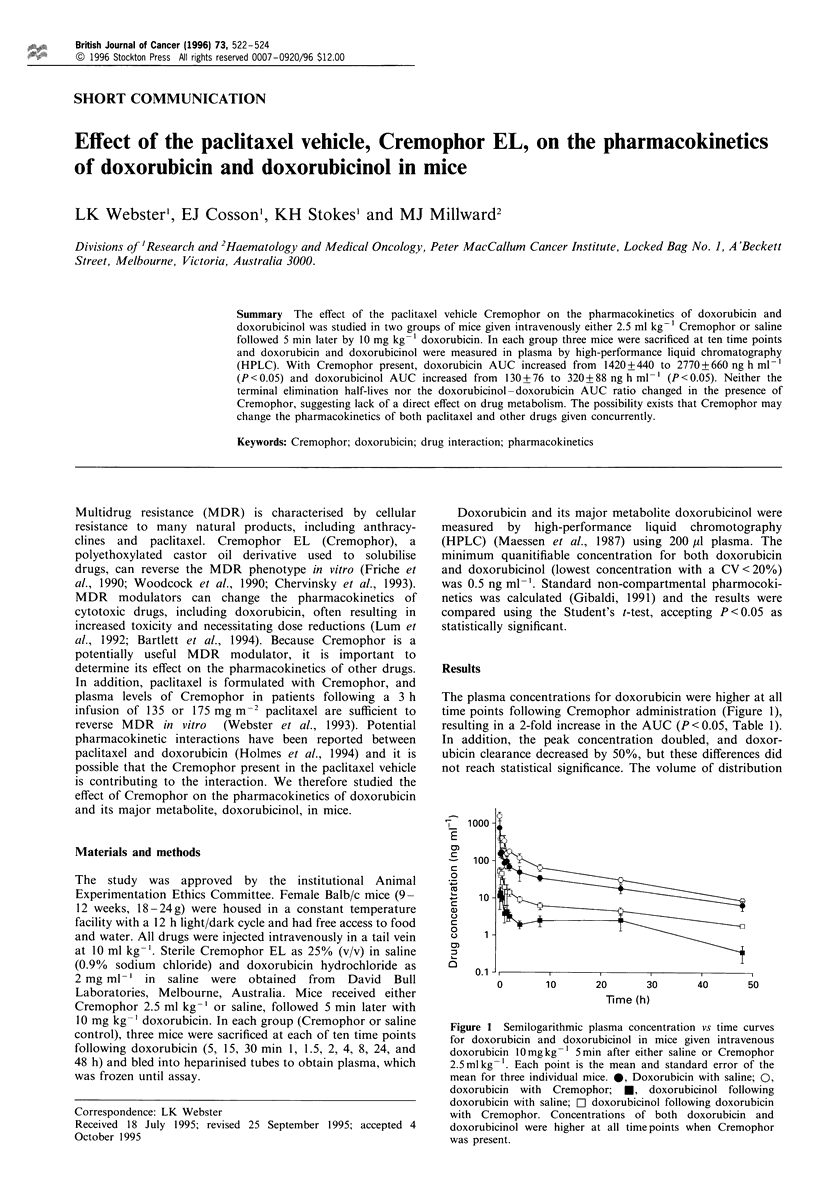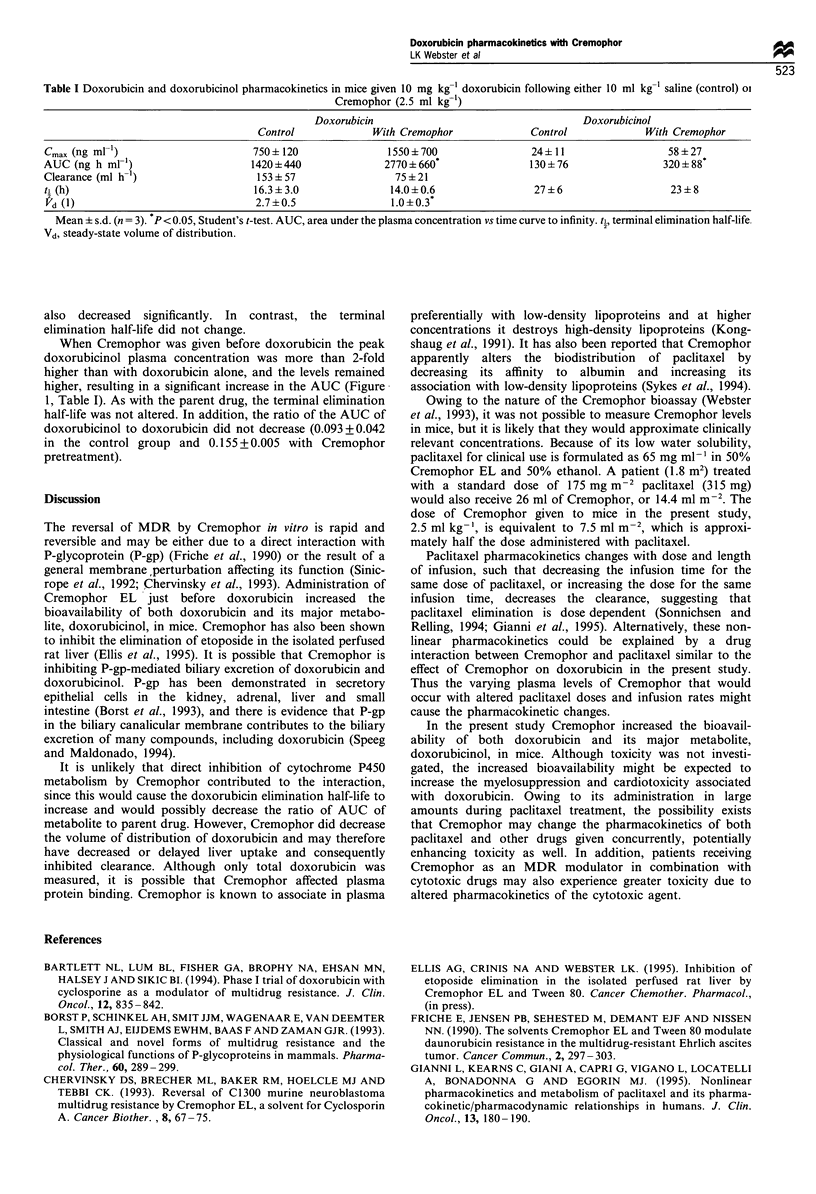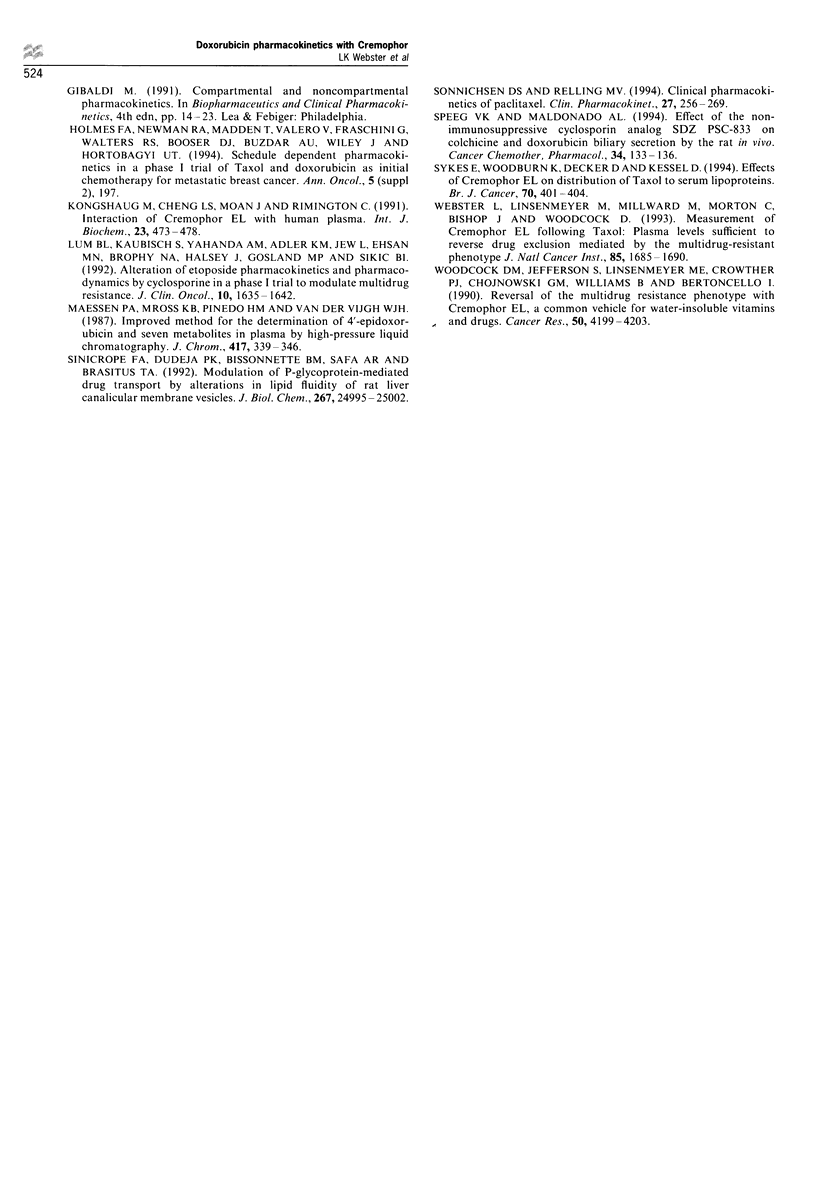# Effect of the paclitaxel vehicle, Cremophor EL, on the pharmacokinetics of doxorubicin and doxorubicinol in mice.

**DOI:** 10.1038/bjc.1996.90

**Published:** 1996-02

**Authors:** L. K. Webster, E. J. Cosson, K. H. Stokes, M. J. Millward

**Affiliations:** Division of Research, Peter MacCallum Cancer Institute, Melbourne, Victoria, Australia.

## Abstract

The effect of the paclitaxel vehicle Cremophor on the pharmacokinetics of doxorubicin and doxorubicinol was studied in two groups of mice given intravenously either 2.5 ml kg-1 Cremophor or saline followed 5 min later by 10 mg kg-1 doxorubicin. In each group three mice were sacrificed at ten time points and doxorubicin and doxorubicinol were measured in plasma by high-performance liquid chromatography (HPLC). With Cremophor present, doxorubicin AUC increased from 1420+/-440 to 2770+/-660 ng h ml(-1) (P<0.05) and doxorubicinol AUC increased from 130+/-76 to 320+/-88 ng h ml(-1) (p<0.05). Neither the terminal elimination half-lives nor the doxorubicinol-doxorubicin AUC ratio changed in the presence of Cremophor, suggesting a lack of a direct effect on drug metabolism. The possibility exists the Cremophor may change the pharmacokinetics of both paclitaxel and other drugs given concurrently.


**British Journal of Cancer (1996) 73, 522–524**
© 1996 Stockton Press All rights reserved 0007–0920/96 $12.00

SHORT COMMUNICATION

### Effect of the paclitaxel vehicle, Cremophor EL, on the pharmacokinetics of doxorubicin and doxorubicinol in mice

LK Webster[1], EJ Cosson[1], KH Stokes[1] and MJ Millward[2]

*Divisions of [1]Research and [2]Haematology and Medical Oncology, Peter MacCallum Cancer Institute, Locked Bag No. 1, A'Beckett Street, Melbourne, Victoria, Australia 3000.*

**Summary** The effect of the paclitaxel vehicle Cremophor on the pharmacokinetics of doxorubicin and doxorubicinol was studied in two groups of mice given intravenously either 2.5 ml $kg^{-1}$ Cremophor or saline followed 5 min later by 10 mg $kg^{-1}$ doxorubicin. In each group three mice were sacrificed at ten time points and doxorubicin and doxorubicinol were measured in plasma by high-performance liquid chromatography (HPLC). With Cremophor present, doxorubicin AUC increased from $1420 \pm 440$ to $2770 \pm 660$ ng h $ml^{-1}$ ($P < 0.05$) and doxorubicinol AUC increased from $130 \pm 76$ to $320 \pm 88$ ng h $ml^{-1}$ ($P < 0.05$). Neither the terminal elimination half-lives nor the doxorubicinol–doxorubicin AUC ratio changed in the presence of Cremophor, suggesting lack of a direct effect on drug metabolism. The possibility exists that Cremophor may change the pharmacokinetics of both paclitaxel and other drugs given concurrently.

**Keywords:** Cremophor; doxorubicin; drug interaction; pharmacokinetics

Multidrug resistance (MDR) is characterised by cellular resistance to many natural products, including anthracyclines and paclitaxel. Cremophor EL (Cremophor), a polyethoxylated castor oil derivative used to solubilise drugs, can reverse the MDR phenotype *in vitro* (Friche *et al.*, 1990; Woodcock *et al.*, 1990; Chervinsky *et al.*, 1993). MDR modulators can change the pharmacokinetics of cytotoxic drugs, including doxorubicin, often resulting in increased toxicity and necessitating dose reductions (Lum *et al.*, 1992; Bartlett *et al.*, 1994). Because Cremophor is a potentially useful MDR modulator, it is important to determine its effect on the pharmacokinetics of other drugs. In addition, paclitaxel is formulated with Cremophor, and plasma levels of Cremophor in patients following a 3 h infusion of 135 or 175 mg $m^{-2}$ paclitaxel are sufficient to reverse MDR *in vitro* (Webster *et al.*, 1993). Potential pharmacokinetic interactions have been reported between paclitaxel and doxorubicin (Holmes *et al.*, 1994) and it is possible that the Cremophor present in the paclitaxel vehicle is contributing to the interaction. We therefore studied the effect of Cremophor on the pharmacokinetics of doxorubicin and its major metabolite, doxorubicinol, in mice.

### Materials and methods

The study was approved by the institutional Animal Experimentation Ethics Committee. Female Balb/c mice (9–12 weeks, 18–24 g) were housed in a constant temperature facility with a 12 h light/dark cycle and had free access to food and water. All drugs were injected intravenously in a tail vein at 10 ml $kg^{-1}$. Sterile Cremophor EL as 25% (v/v) in saline (0.9% sodium chloride) and doxorubicin hydrochloride as 2 mg $ml^{-1}$ in saline were obtained from David Bull Laboratories, Melbourne, Australia. Mice received either Cremophor 2.5 ml $kg^{-1}$ or saline, followed 5 min later with 10 mg $kg^{-1}$ doxorubicin. In each group (Cremophor or saline control), three mice were sacrificed at each of ten time points following doxorubicin (5, 15, 30 min 1, 1.5, 2, 4, 8, 24, and 48 h) and bled into heparinised tubes to obtain plasma, which was frozen until assay.

Doxorubicin and its major metabolite doxorubicinol were measured by high-performance liquid chromotography (HPLC) (Maessen *et al.*, 1987) using 200 μl plasma. The minimum quanitifiable concentration for both doxorubicin and doxorubicinol (lowest concentration with a CV < 20%) was 0.5 ng $ml^{-1}$. Standard non-compartmental pharmocokinetics was calculated (Gibaldi, 1991) and the results were compared using the Student's *t*-test, accepting $P < 0.05$ as statistically significant.

### Results

The plasma concentrations for doxorubicin were higher at all time points following Cremophor administration (Figure 1), resulting in a 2-fold increase in the AUC ($P < 0.05$, Table 1). In addition, the peak concentration doubled, and doxorubicin clearance decreased by 50%, but these differences did not reach statistical significance. The volume of distribution

**Figure 1** Semilogarithmic plasma concentration *vs* time curves for doxorubicin and doxorubicinol in mice given intravenous doxorubicin 10 mg $kg^{-1}$ 5 min after either saline or Cremophor 2.5 ml $kg^{-1}$. Each point is the mean and standard error of the mean for three individual mice. ●, Doxorubicin with saline; ○, doxorubicin with Cremophor; ■, doxorubicinol following doxorubicin with saline; □ doxorubicinol following doxorubicin with Cremophor. Concentrations of both doxorubicin and doxorubicinol were higher at all time points when Cremophor was present.

Correspondence: LK Webster
Received 18 July 1995; revised 25 September 1995; accepted 4 October 1995

Table I Doxorubicin and doxorubicinol pharmacokinetics in mice given 10 mg $kg^{-1}$ doxorubicin following either 10 ml $kg^{-1}$ saline (control) or Cremophor (2.5 ml $kg^{-1}$)

| | *Doxorubicin* | | *Doxorubicinol* | |
|---|---|---|---|---|
| | *Control* | *With Cremophor* | *Control* | *With Cremophor* |
| $C_{max}$ (ng $ml^{-1}$) | 750 ± 120 | 1550 ± 700 | 24 ± 11 | 58 ± 27 |
| AUC (ng h $ml^{-1}$) | 1420 ± 440 | 2770 ± 660* | 130 ± 76 | 320 ± 88* |
| Clearance (ml $h^{-1}$) | 153 ± 57 | 75 ± 21 | | |
| $t_{\frac{1}{2}}$ (h) | 16.3 ± 3.0 | 14.0 ± 0.6 | 27 ± 6 | 23 ± 8 |
| $V_d$ (l) | 2.7 ± 0.5 | 1.0 ± 0.3* | | |

Mean ± s.d. ($n = 3$). *$P < 0.05$, Student's $t$-test. AUC, area under the plasma concentration *vs* time curve to infinity. $t_{\frac{1}{2}}$, terminal elimination half-life. $V_d$, steady-state volume of distribution.

also decreased significantly. In contrast, the terminal elimination half-life did not change.

When Cremophor was given before doxorubicin the peak doxorubicinol plasma concentration was more than 2-fold higher than with doxorubicin alone, and the levels remained higher, resulting in a significant increase in the AUC (Figure 1, Table I). As with the parent drug, the terminal elimination half-life was not altered. In addition, the ratio of the AUC of doxorubicinol to doxorubicin did not decrease (0.093 ± 0.042 in the control group and 0.155 ± 0.005 with Cremophor pretreatment).

### Discussion

The reversal of MDR by Cremophor *in vitro* is rapid and reversible and may be either due to a direct interaction with P-glycoprotein (P-gp) (Friche *et al.*, 1990) or the result of a general membrane perturbation affecting its function (Sinicrope *et al.*, 1992; Chervinsky *et al.*, 1993). Administration of Cremophor EL just before doxorubicin increased the bioavailability of both doxorubicin and its major metabolite, doxorubicinol, in mice. Cremophor has also been shown to inhibit the elimination of etoposide in the isolated perfused rat liver (Ellis *et al.*, 1995). It is possible that Cremophor is inhibiting P-gp-mediated biliary excretion of doxorubicin and doxorubicinol. P-gp has been demonstrated in secretory epithelial cells in the kidney, adrenal, liver and small intestine (Borst *et al.*, 1993), and there is evidence that P-gp in the biliary canalicular membrane contributes to the biliary excretion of many compounds, including doxorubicin (Speeg and Maldonado, 1994).

It is unlikely that direct inhibition of cytochrome P450 metabolism by Cremophor contributed to the interaction, since this would cause the doxorubicin elimination half-life to increase and would possibly decrease the ratio of AUC of metabolite to parent drug. However, Cremophor did decrease the volume of distribution of doxorubicin and may therefore have decreased or delayed liver uptake and consequently inhibited clearance. Although only total doxorubicin was measured, it is possible that Cremophor affected plasma protein binding. Cremophor is known to associate in plasma preferentially with low-density lipoproteins and at higher concentrations it destroys high-density lipoproteins (Kongshaug *et al.*, 1991). It has also been reported that Cremophor apparently alters the biodistribution of paclitaxel by decreasing its affinity to albumin and increasing its association with low-density lipoproteins (Sykes *et al.*, 1994).

Owing to the nature of the Cremophor bioassay (Webster *et al.*, 1993), it was not possible to measure Cremophor levels in mice, but it is likely that they would approximate clinically relevant concentrations. Because of its low water solubility, paclitaxel for clinical use is formulated as 65 mg $ml^{-1}$ in 50% Cremophor EL and 50% ethanol. A patient (1.8 $m^2$) treated with a standard dose of 175 mg $m^{-2}$ paclitaxel (315 mg) would also receive 26 ml of Cremophor, or 14.4 ml $m^{-2}$. The dose of Cremophor given to mice in the present study, 2.5 ml $kg^{-1}$, is equivalent to 7.5 ml $m^{-2}$, which is approximately half the dose administered with paclitaxel.

Paclitaxel pharmacokinetics changes with dose and length of infusion, such that decreasing the infusion time for the same dose of paclitaxel, or increasing the dose for the same infusion time, decreases the clearance, suggesting that paclitaxel elimination is dose dependent (Sonnichsen and Relling, 1994; Gianni *et al.*, 1995). Alternatively, these non-linear pharmacokinetics could be explained by a drug interaction between Cremophor and paclitaxel similar to the effect of Cremophor on doxorubicin in the present study. Thus the varying plasma levels of Cremophor that would occur with altered paclitaxel doses and infusion rates might cause the pharmacokinetic changes.

In the present study Cremophor increased the bioavailability of both doxorubicin and its major metabolite, doxorubicinol, in mice. Although toxicity was not investigated, the increased bioavailability might be expected to increase the myelosuppression and cardiotoxicity associated with doxorubicin. Owing to its administration in large amounts during paclitaxel treatment, the possibility exists that Cremophor may change the pharmacokinetics of both paclitaxel and other drugs given concurrently, potentially enhancing toxicity as well. In addition, patients receiving Cremophor as an MDR modulator in combination with cytotoxic drugs may also experience greater toxicity due to altered pharmacokinetics of the cytotoxic agent.

### References

BARTLETT NL, LUM BL, FISHER GA, BROPHY NA, EHSAN MN, HALSEY J AND SIKIC BI. (1994). Phase I trial of doxorubicin with cyclosporine as a modulator of multidrug resistance. *J. Clin. Oncol.*, **12**, 835–842.

BORST P, SCHINKEL AH, SMIT JJM, WAGENAAR E, VAN DEEMTER L, SMITH AJ, EIJDEMS EWHM, BAAS F AND ZAMAN GJR. (1993). Classical and novel forms of multidrug resistance and the physiological functions of P-glycoproteins in mammals. *Pharmacol. Ther.*, **60**, 289–299.

CHERVINSKY DS, BRECHER ML, BAKER RM, HOELCLE MJ AND TEBBI CK. (1993). Reversal of C1300 murine neuroblastoma multidrug resistance by Cremophor EL, a solvent for Cyclosporin A. *Cancer Biother.*, **8**, 67–75.

ELLIS AG, CRINIS NA AND WEBSTER LK. (1995). Inhibition of etoposide elimination in the isolated perfused rat liver by Cremophor EL and Tween 80. *Cancer Chemother. Pharmacol.*, (in press).

FRICHE E, JENSEN PB, SEHESTED M, DEMANT EJF AND NISSEN NN. (1990). The solvents Cremophor EL and Tween 80 modulate daunorubicin resistance in the multidrug-resistant Ehrlich ascites tumor. *Cancer Commun.*, **2**, 297–303.

GIANNI L, KEARNS C, GIANI A, CAPRI G, VIGANO L, LOCATELLI A, BONADONNA G AND EGORIN MJ. (1995). Nonlinear pharmacokinetics and metabolism of paclitaxel and its pharmacokinetic/pharmacodynamic relationships in humans. *J. Clin. Oncol.*, **13**, 180–190.

GIBALDI M. (1991). Compartmental and noncompartmental pharmacokinetics. In *Biopharmaceutics and Clinical Pharmacokinetics*, 4th edn, pp. 14–23. Lea & Febiger: Philadelphia.

HOLMES FA, NEWMAN RA, MADDEN T, VALERO V, FRASCHINI G, WALTERS RS, BOOSER DJ, BUZDAR AU, WILEY J AND HORTOBAGYI UT. (1994). Schedule dependent pharmacokinetics in a phase I trial of Taxol and doxorubicin as initial chemotherapy for metastatic breast cancer. *Ann. Oncol.*, **5** (suppl 2), 197.

KONGSHAUG M, CHENG LS, MOAN J AND RIMINGTON C. (1991). Interaction of Cremophor EL with human plasma. *Int. J. Biochem.*, **23,** 473–478.

LUM BL, KAUBISCH S, YAHANDA AM, ADLER KM, JEW L, EHSAN MN, BROPHY NA, HALSEY J, GOSLAND MP AND SIKIC BI. (1992). Alteration of etoposide pharmacokinetics and pharmacodynamics by cyclosporine in a phase I trial to modulate multidrug resistance. *J. Clin. Oncol.*, **10,** 1635–1642.

MAESSEN PA, MROSS KB, PINEDO HM AND VAN DER VIJGH WJH. (1987). Improved method for the determination of 4′-epidoxorubicin and seven metabolites in plasma by high-pressure liquid chromatography. *J. Chrom.*, **417,** 339–346.

SINICROPE FA, DUDEJA PK, BISSONNETTE BM, SAFA AR AND BRASITUS TA. (1992). Modulation of P-glycoprotein-mediated drug transport by alterations in lipid fluidity of rat liver canalicular membrane vesicles. *J. Biol. Chem.*, **267,** 24995–25002.

SONNICHSEN DS AND RELLING MV. (1994). Clinical pharmacokinetics of paclitaxel. *Clin. Pharmacokinet.*, **27,** 256–269.

SPEEG VK AND MALDONADO AL. (1994). Effect of the non-immunosuppressive cyclosporin analog SDZ PSC-833 on colchicine and doxorubicin biliary secretion by the rat *in vivo*. *Cancer Chemother, Pharmacol.*, **34,** 133–136.

SYKES E, WOODBURN K, DECKER D AND KESSEL D. (1994). Effects of Cremophor EL on distribution of Taxol to serum lipoproteins. *Br. J. Cancer*, **70,** 401–404.

WEBSTER L, LINSENMEYER M, MILLWARD M, MORTON C, BISHOP J AND WOODCOCK D. (1993). Measurement of Cremophor EL following Taxol: Plasma levels sufficient to reverse drug exclusion mediated by the multidrug-resistant phenotype *J. Natl Cancer Inst.*, **85,** 1685–1690.

WOODCOCK DM, JEFFERSON S, LINSENMEYER ME, CROWTHER PJ, CHOJNOWSKI GM, WILLIAMS B AND BERTONCELLO I. (1990). Reversal of the multidrug resistance phenotype with Cremophor EL, a common vehicle for water-insoluble vitamins and drugs. *Cancer Res.*, **50,** 4199–4203.